# Supplementary figures and images for: Assessment of drug-related problems among breast cancer patients in a cancer specialty center in Nepal
Source: PLoS One. 2025 Oct 17;20(10):e0334703. doi: 10.1371/journal.pone.0334703 (PMC12533890; doi:10.1371/journal.pone.0334703)

**Date according to Gregorian Calendar: 25/04/2024**


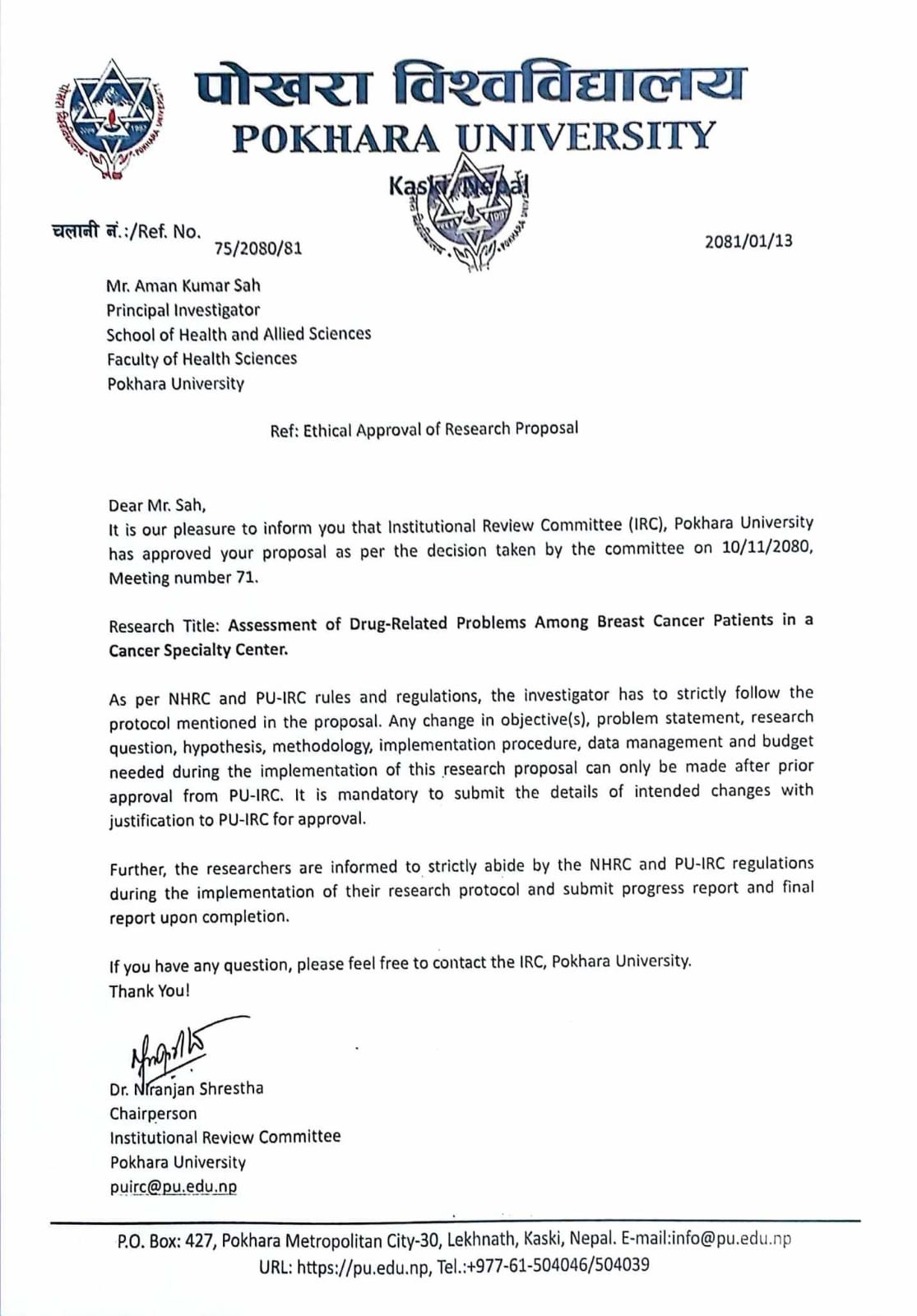

Supplement: S2 File — (DOCX) [file pone.0334703.s002.docx]
