# Supplementary material for: Assessment of drug-related problems among breast cancer patients in a cancer specialty center in Nepal
Source: PLoS One. 2025 Oct 17;20(10):e0334703. doi: 10.1371/journal.pone.0334703 (PMC12533890; doi:10.1371/journal.pone.0334703)
Supplement: Attachment — Submitted filename: Response to Reviewers.docx [file pone.0334703.s005.docx]

Reviewer #1: • The title is clear and concise, accurately reflecting the study's focus on drug-related problems (DRPs) among breast cancer patients in Nepal.

**Response to reviewer 1 comment:** Thank you for the general positive assessment of the paper.

The abstract provides a good summary of the study, including the background, methods, results, and conclusion. However, it could benefit from a brief mention of the study's limitations.

**Response to reviewer 1 comment:** Thank you for your suggestion. We have now updated our abstract with the limitation too amending in the conclusion section. **[Addressed in line numbers 43-46, Page 3]**

The introduction could be enhanced by providing more detailed information on previous studies related to DRPs in breast cancer patients, particularly in similar settings.

**Response to reviewer 1 comment:** We have now added literatures relating to breast cancer along with the context of Drug related problems in the introduction section. **[Addressed in line numbers 72-81, Page numbers 4-5]**

The sample size calculation is explained, but the rationale for choosing a 50% prevalence rate could be clarified. Additionally, more information on how patient interviews were conducted and how data accuracy was ensured would be beneficial.

**Response to reviewer 1 comment:** Thank you highlighting this issue. We chose the 50% prevlance rate as there was no specific prevalence data regarding the Drug related problems within the context of Nepal specific to breast carcinoma patients. Hence, to create a generalized data collection, we chose the 50% through our supervison with our statitican and literature review. We have now added information regarding patient interview, data accuracy in methodology section. **[Addressed in Line numbers 127-129, 138-143, 145-160 Page numbers 7-8]**

The rationale for choosing a cross-sectional design could be elaborated. Additionally, more details about the hospital's patient population and its relevance to the study could provide better context.

**Response to reviewer 1 comment:** Thank you for underlining this deficiency. We have now added the context related to cross-sectional design and also included information regarding hospital’s patient population. **[Addressed in Line numbers 94-97, 101-111, Page numbers 5-6]**

The manuscript could benefit from a more detailed description of how patient confidentiality was maintained and any measures taken to ensure ethical standards were upheld throughout the study.

**Response to reviewer 1 comment:** We have now added this information within a separate section as “Ethical Consideration”. **[Addressed in Line numbers 167-169, Page number 9]**

The exclusion criteria are not mentioned and should be included to provide a complete understanding of the study population.

**Response to reviewer 1 comment:** We have now mentioned the exclusion criteria within the sections “inclusion and exclusion criteria”. **[Addressed in Line numbers 106-111, Page number 6]**

More details on how patient interviews were conducted, including the interview process, duration, and any training provided to the interviewers, would enhance the transparency of the data collection process. Additionally, the manuscript should describe how data accuracy and consistency were ensured.

**Response to reviewer 1 comment:** We have now added information regarding the patient interview and related information. **[Addressed in Line numbers 145-160, Page number 8]**

The manuscript could benefit from a more detailed explanation of the statistical analysis, including any software used and the rationale for choosing specific statistical tests. Additionally, the manuscript should discuss how missing data were handled and any sensitivity analyses performed.

**Response to reviewer 1 comment:** Thank you for underlining this deficiency. We have now added information within the data analysis section. For those process not conducted we have included in the limitation section. In case the data was missing, it was not included (falls within the exclusion critiera) as it interfered with the DRPs findings. **[Addressed in Line numbers 182-195, 155-160, 352-361 Page numbers 10, 8 and 32]**

The manuscript should provide more details on the criteria used by the oncologists to verify DRPs and how discrepancies between the two oncologists were resolved.

**Response to reviewer 1 comment:** We have now added information regarding this issue. discrepancies regarding DRPs between the oncologists were recorded then it was further discussed in the hospital tumor board meetings. The decision of tumor board was the final one. **[Addressed in Line numbers 159-161, Page number 8 and Fig 1]**

The Methods section should explicitly mention potential limitations related to the study design, data collection, and analysis. For example, the use of a single center and the potential for selection bias should be discussed.

**Response to reviewer 1 comment:** Thank you for underlining this deficiency. We have highlighted this information in our study limitation. **[Addressed in Line numbers 351-361, Page number 32]**

To enhance the rigor and transparency of the study, the manuscript should provide more detailed justifications for methodological choices, describe the data collection and verification processes in greater detail, and explicitly discuss potential limitations.

**Response to reviewer 1 comment:** We have discuseed the methodological chocices, data collection procedure, potential limitation with respect to the above comments too. **[Addressed in Line numbers 101-104, 145-160, 351-361 Page numbers 6, 8, 32]**

The results section could be improved by providing more detailed statistical analysis, including confidence intervals for key findings. Additionally, a more in-depth discussion of the types of DRPs and their potential impact on patient outcomes would be valuable.

**Response to reviewer 1 comment:** Our study didn’t included the confidence interval for key findings. We have included this in our limitation too. We have now added information to the discussion of DRPs and their potential impact too in the discussion section. **[Addressed in Line numbers 297- 307, 351-360 Page number 30, 32]**

The discussion could be strengthened by exploring potential interventions to reduce DRPs and improve patient outcomes. Additionally, the study's limitations should be discussed in more detail, including potential biases and the generalizability of the findings.

**Response to reviewer 1 comment:** We have now added the interventional context with the discussion section. We have also added information in the limitation section. **[Addresssed in Line numbers 327-349, 351-353, Page 31-32]**

The conclusion could be enhanced by providing specific recommendations for future research and clinical practice.

**Response to reviewer 1 comment:** We have now added the recommendations in the end of the conclusion. **[Addressed in Line numbers 357-361, Page number 32].**

Ensure all references are formatted consistently according to the journal's guidelines.

**Response to reviewer 1 comment:** We have now formatted all the references according to the journal’s guidelines i.e. Vancouver as mentioned in the journal official website. [<https://journals.plos.org/plosone/s/submission-guidelines>]. **[Addressed in Line numbers 372-518, Page number 34-37].**

To enhance the manuscript, consider providing more detailed statistical analysis, discussing the study's limitations more thoroughly, and offering specific recommendations for clinical practice and future research.

**Response to reviewer 1 comment:** Thank you for the suggestion. We have added information relating to methodological context, statistical analysis, limitation and recommendation for future research. **[Addressed in Line numbers 351-361, Page number 32]**

Reviewer #2: This is an interesting study and the authors have done a good job on its execution. Please find some of my comments for better outcome for manuscript:

**Response to reviewer 2 comment:** Thank you for general positive assessment of the paper. We have addressed each comments with their responses.

Introduction section:

-The whole manuscript may be revised for clarity and flow. Redundant concepts/ideas need to be revised (Line 66-68 and 70-72: pretty much share the same idea of patients being susceptible and a lack of local research).

**Response to reviewer 2 comment:** Thank you for underlining this redundant issue. We have also revised several sentences for clarity and flow. We have also removed the redundant sensteces.

-Line 69-70: Consider reframing this sentence. The sentence is phrased awkwardly as of now.
-The authors may emphasize the rationale of this study more in the last section of the Introduction.

**Response to reviewer 2 comment:** We have now removed the sentences and added context relating to DRPs from other literatures along with focus on the rationale of the study. Line number 81-82 sentence is removed. **[Addressed in line numbers 74-81, 82-89, Page number 5]**

Method Section:

-Since the sample size is calculated, a clear justification for the use of the purposive sampling method (non-probabilistic sampling method) needs to be made. Was it because of a smaller population group, feasibility issues, or was it something else?

**Response to reviewer 2 comment:** Thank you for the suggestion. We have included the information as “Since the sample size calucated was very less and the prevalence and patient coming to hospital for breast carcima care is quite less, it limited us in using random samapling technique. We chose the purposive sampling as it ensured proper respresntation of breast cancer patients with related characteristics increasing the reliabitliy of the findings. Practical constraints such as time and logistic factors were also barrier for that led us to choose the purposing sampling for the study” in the section Sampling size and sampling technique. **[Addressed in Line numbers 127-132, Page number 7]**

-DRP identification by two individuals was a great approach, but how was any discrepancy among them resolved? Adding some information about the management of conflicts in case of occurrence would add value.

**Response to reviewer 2 comment:** We have now added information regarding this issue. discrepancies regarding DRPs between the oncologists were recorded then it was further discussed in the hospital tumor board meetings. The decision of tumor board was the final one. **[Addressed in Line numbers 159-161, Page number 8, and Fig 1]**

Explain the terms used in the formula for sample size calculations. It is not well formulated in the current version.

**Response to reviewer 2 comment:** We have now explained the terms used in the sample size calculation formula. **[Addressed in Line numbers 115-125, Page number 6-7]**

If possible, information on which variables (sociodemographic or clinical variables) were tested for association with DRPs in the method section itself would add better flow to the reader.

**Response to reviewer 2 comment:**We have now added the information on which variables were tested for association with DRPs. **[Addressed in Line numbers 175-180, Page number 9]**

Results and discussion:

-The results and discussion sections are well formulated. Make sure the percentages used in the brackets are aligned with the one mentioned in the result writing and tables.

**Response to reviewer 2 comment:** We have now checked for the percentages used in the discussion and aligned it with the result section.

-Line 135: 91 or 92 patients?

**Response to reviewer 2 comment:** Thank you for underlining the confusion. Total breast cancer patient enrolled in the study were 92, among which in 91 patient 104 DRPs was observed. In this sentence, 91 breast cancer patients is ok.

Conclusion:

-Line 245: May be rephrased by removing the word ‘significant’ since this is not regarding any statistical test, but rather just the frequency.

**Response to reviewer 2 comment:** We have now removed the word ‘significant’.
